# Comparison of propofol-ketamine and propofol-fentanyl combinations for sedation in patients undergoing gastrointestinal endoscopy: a randomized clinical trial

**DOI:** 10.1016/j.bjane.2024.844561

**Published:** 2024-09-19

**Authors:** Milena Vasconcelos Falcão, João Nogueira Neto, Ed Carlos Rey Moura, Caio Márcio Barros Oliveira, Rodrigo Pinto Diniz, Almir Vieira Dibai-Filho, Plínio da Cunha Leal

**Affiliations:** Universidade Federal do Maranhão (UFMA), São Luís, MA, Brazil

Dear Editor,

Colonoscopy is a globally performed procedure, playing a crucial role in diagnosing and treating colon diseases, and significantly reducing colorectal cancer risk in symptomatic patients and those undergoing screening. Pain during colonoscopy arises from mesenteric traction maneuvers and distension of the colonic lumen, making adequate sedation and analgesia essential. Regarding the ketamine-propofol combination, the synergistic effects of these drugs can counterbalance their individual disadvantages; ketamine's sympathomimetic properties mitigate the hypotension and respiratory depression caused by propofol, while propofol reduces ketamine's adverse gastrointestinal effects.^1^

This randomized, double-blind clinical trial carried out between May 2022 and February 2023, conducted at Hospital São Domingos, São Luís, Maranhão, Brazil, aimed to evaluate the sedative efficacy by two different sedation regimens (Propofol + Ketamine vs. Propofol + Fentanyl) in patients undergoing elective Colonoscopy or Colonoscopy + Endoscopy. The study received ethical approval from the Research Ethics Committee (CAAE: 58003522.0.0000.5085) and was registered in the Brazilian Registry of Clinical Trials (REBEC) (RBR-4mvny8s).

Patients aged 18 to 65 years, classified as American Society of Anesthesiologists (ASA) physical status I or II, and scheduled for elective Colonoscopy (CLN) or bidirectional endoscopy (colonoscopy and upper endoscopy) were included. The sample size was calculated with a difference of at least 30% between the two groups regarding patient dropout or complications, considering an alpha error of 5% and a statistical power of 80%, 40 patients were allocated to each group. The primary objective was to assess sedation safety and effectiveness in achieving hemodynamic stability, with secondary objectives being the analysis of procedure duration, propofol doses, and satisfaction levels of both patients and anesthesiologists. Data analysis was performed using SPSS V.26. Categorical data were presented in absolute and relative frequency, and numerical data in mean and standard deviation or median and range. The Chi-Square, in categorical variables, *t-*test or Mann-Whitney in continuous variables, according to normality, verified by Shapiro-Wilk, were used to compare groups.

In the PF group (Propofol + Fentanyl), sedation and analgesia were performed with propofol 1 mg.kg^-1^ and fentanyl 0.25 µg.kg^-1^, and in the PK group (Propofol + Ketamine), sedation and analgesia were performed with propofol 1 mg.kg^-1^ and ketamine 0.25 mg.kg^-1^.

Demographic, clinical and anthropometry data were collected before the procedure. Heart Rate (HR), Respiratory Rate (RR), Peripheral O_2_ Saturation (SpO_2_), Systolic Blood Pressure (SBP) and Diastolic Blood Pressure (DBP) were collected at the beginning and at 5, 10, 15, 20 and 30 minutes throughout the procedure. Procedure duration, sedation-related complications (including Systolic Blood Pressure [SBP < 85 mmHg] or Diastolic Blood Pressure [DBP < 50 mmHg], Mean Arterial Pressure [MAP > 20 mmHg], Heart Rate [HR < 55 bpm], anesthetic recovery (period after the end of the procedure until a score of 2 in the Ramsay Sedation Scale is reached), Ramsay Sedation Scale (RSS), hospital discharge and Visual Analog Scale of Satisfaction (1 to 10, 10 being the best satisfaction rate) were also recorded after the procedure.

The PK group included 40 patients, and 40 in the PF group. The social and clinical profiles showed no statistical difference. A statistically significant higher incidence of cardiovascular complications was observed in the PF group (62.5%, *p* = 0.025). The PK group had a significantly shorter discharge time (30.0 ± 8.7 minutes, *p* = 0.019) (Table 1).

**Table 1:** Social and clinical profile of patients undergoing elective colonoscopy or Colonoscopy + Endoscopy procedures with sedation performed with Propofol-Fentanyl (n = 40) or Propofol-Ketamine (n = 40).

Variables	PF	PK	*p*-value
**Social and clinical profile**			
Age range (years) – Md ± Sd	52.0 ± 12.3	53.7 ± 9.8	0.508^a^
Gender – n (%)			
Male	10 (25.0)	15 (37.5)	0.228^b^
Female	30 (75.0)	25 (62.5)	
Comorbidities			
Yes	31 (77.5)	28 (70.0)	0.446^b^
No	9 (22.5)	12 (30.0)	
Anthropometry			
Weight (kg) – Md ± Sd	70.5±11.5	73.3±13.4	0.319^a^
BMI (kg.m^-2^) – Md ± Sd	26.8±3.9	27.6±4.1	0.337^a^
Reason – n (%)			
Screening	19 (47.5)	16 (40.0)	0.499^b^
Diagnosis	21 (52.5)	24 (60.0)	
Procedure – n (%)			
CLN	18 (45.0)	26 (65.0)	0.072^b^
Bidirectional endoscopy	22 (55.0)	14 (35.0)	
Perioperative data			
Cardiovascular complications – n (%)			
Yes	25 (62.5)	15 (37.5)	***0.025***^b^
No	15 (37.5)	25 (62.5)	
SBP < 85, DBP < 50 or ∆ da MAP > 20 – n (%)			
Yes	23 (57.5)	15 (37.5)	0.0732^b^
No	17 (42.5)	25 (62.5)	
Procedure duration (min) – Median (Min‒Max)	31 (8‒59)	27.5 (9‒52)	0.470^c^
Recovery duration to hospital discharge (min) – Md ± Dp	34.8±9.1	30.0±8.7	***0.019***^a^
Initial dose of propofol (mg) – Md ± Sd	73.3±13.0	70.5±11.5	0.316^a^
Additional dose of propofol (mg) – Md ± Sd	138.6±88	117.2±102.5	0.319^a^
Recovery time (until RSS2) – Median (Min‒Max)	10 (10‒20)	10 (10‒20)	0.628^c^
Patient satisfaction – Md ± Sd	10.0±0.0	10.0±0.0	1.000^a^
Anesthesiologist satisfaction – Md ± Sd	9.2±1.3	9.1±1.2	0.721^a^

PF, Propofol + Fentanyl; PK, Propofol + ketamine; CLN, Colonoscopy; Comorbidities: Obesity, Hypertension, Diabetes mellitus, Hypothyroidism; SBP, Systolic Blood Pressure; DBP, Diastolic Blood Pressure; MAP, Mean Arterial Pressure; RSS, Ramsay Sedation Scale; Md ± Sd, Mean ± Standard deviation.

a Student's *t*-test.

b Chi-Square.

c Mann-Whitney.

Heart Rate (HR) was significantly lower in the PF group at 5, 10, and 15 minutes during the procedure and at 5 and 10 minutes in the recovery room (*p* = 0.012; *p* = 0.003; *p* = 0.026; *p* = 0.008; and *p* = 0.009, respectively), additionally the Respiratory Rate (RR) was significantly lower in the PF group at 5, 10, 15, 20, and 30 minutes during the procedure (*p* = 0.001; *p* = 0.003; *p* = 0.024; *p* = 0.019; and *p* = 0.029, respectively). There was no statistically significant difference in respiratory rate in the recovery room (Material Supp. 1).

SBP was significantly lower in the PF group in all evaluated intervals during the procedure (*p* < 0.05), and DBP was statistically significant at moments 5, 10, 15, 25, and 30 minutes during the procedure (*p* < 0.05), with lower values found in the PF group (Material Supp. 2).

Comparing moments 5, 10, 15, 25, and 30 minutes during the procedure, the PK group had significantly higher MAP values (*p* = 0.001; *p* = 0.000; *p* = 0.000; *p* = 0.016; and *p* = 0.026, respectively). There was no statistical significance between the groups when comparing baseline MAP and recovery period. There was no SBP and DBP baseline statistical significance in the recovery room. Baseline peripheral oxygen saturation (SpO_2_) was significantly lower in the PF group (*p* = 0.029); however, during the procedure and the recovery period, there was no statistical significance (*p* > 0.05) (Material Supp. 3).

The higher incidence of cardiovascular complications in the PF group is consistent with the study by Vettorello et al.,^2^ who found that there is activation of the cardiovagal reflex after administration of low doses of fentanyl.

Consistent with our results, Goh et al^1^ found higher heart rate and higher systolic and mean arterial pressures in the ketamine-propofol group compared to the fentanyl-propofol or propofol alone groups in laryngeal mask insertion procedures in adults.

Hemodynamic stability in the PK group during and after the procedure was consistent with the study by Aydoghan et al,^3^ who found that a propofol and ketamine combination generates improved hemodynamic stability and higher satisfaction compared to propofol alone.

In a study by Goel et al,^4^ the authors demonstrated a significant decrease in SBP when propofol was used alone for induction compared to a propofol and ketamine combination during laryngeal mask insertion in children.

We observed higher respiratory rates in the PK group compared to the PF group, as Mortero et al^5^ demonstrated an increase in the final expiratory PaCO_2_ in patients who received the Propofol + Fentanyl combination and when ketamine was added, the final expiratory PaCO_2_ did not increase.

In conclusion, the propofol-ketamine combination was found to be safe and effective in achieving hemodynamic stability with fewer complications compared to the propofol-fentanyl combination during colonoscopy and bidirectional endoscopy.

## Conflicts of interest

The authors declare no conflicts of interest.

## Appendix Supplementary materials

Image, application 1

## Footnotes

Supplementary material associated with this article can be found in the online version at doi:10.1016/j.bjane.2024.844561.
