# Supplementary material for: Comparison of propofol-ketamine and propofol-fentanyl combinations for sedation in patients undergoing gastrointestinal endoscopy: a randomized clinical trial
Source: Braz J Anesthesiol. 2024 Sep 19;75(1):844561. doi: 10.1016/j.bjane.2024.844561 (PMC11490659; doi:10.1016/j.bjane.2024.844561)

BJAN-D-24-00168_Supplementary Material

**Material Supplement 1** Medians of Heart Rate (HR) and Respiratory Rate (RR) of patients undergoing elective colonoscopy or Colonoscopy + Endoscopy procedures with sedation performed with propofol-fentanyl (n = 40) or propofol-ketamine (n = 40). Mann-Whitney test: * p < 0.05. PF, Propofol + Fentanyl; PK, Propofol + Ketamine.


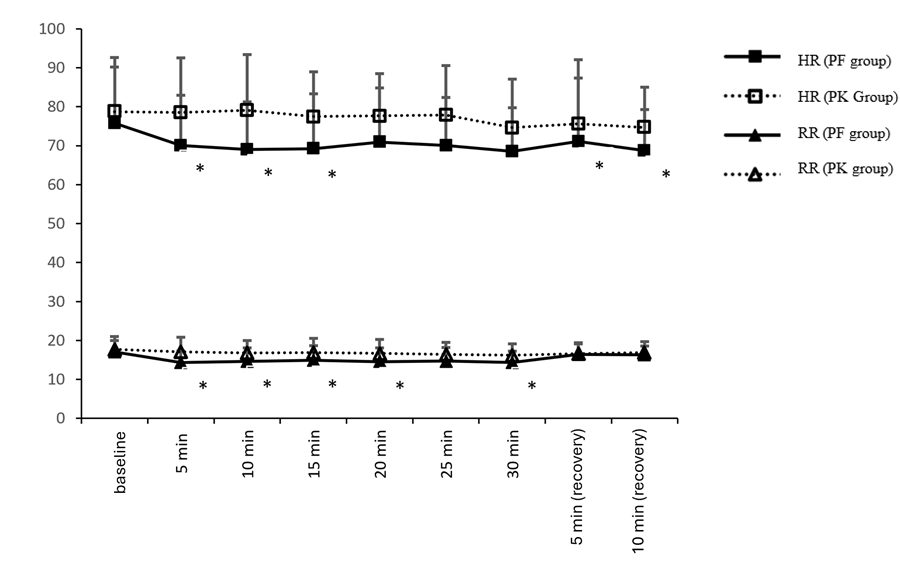


**Material Supplement 2** Medians of Systolic Blood Pressure (SBP) and Diastolic Blood Pressure (DBP) of patients undergoing elective colonoscopy or colonoscopy + endoscopy procedures with sedation performed with propofol-fentanyl (n = 40) or propofol-ketamine (n = 40). Mann-Whitney test: * p < 0.05. PF, Propofol + Fentanyl; PK, Propofol + Ketamine.


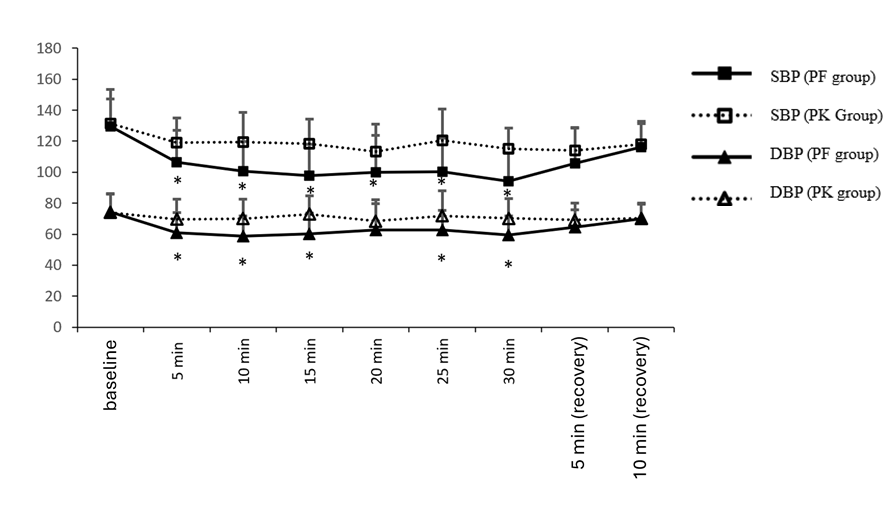


**Material Supplement 3** Medians of the Mean Arterial Pressure (MAP) and Peripheral Oxygen Saturation (SpO_2_) of patients undergoing elective colonoscopy or colonoscopy + endoscopy procedures with sedation performed with propofol-fentanyl (n = 40) or propofol-ketamine (n = 40). Mann-Whitney test: *p < 0.05. PF, Propofol + Fentanyl; PK, Propofol + Ketamine.


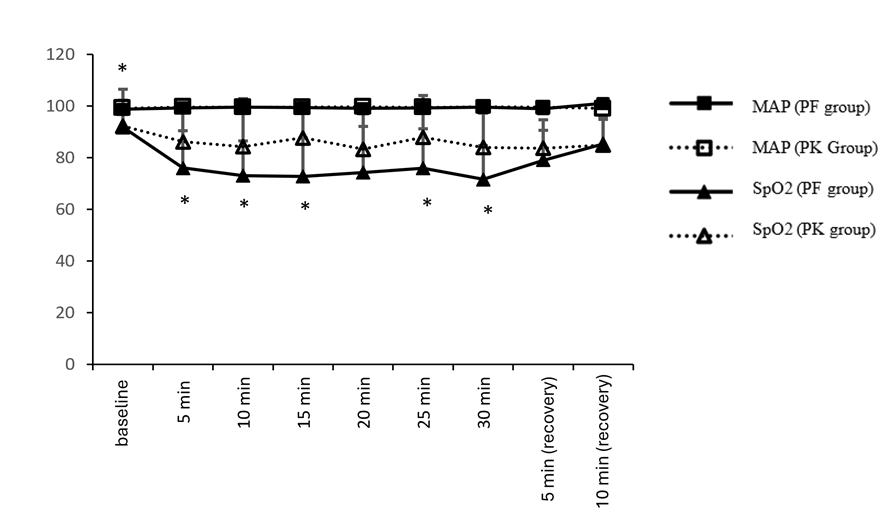

Supplement: Supplementary file 1 [file mmc1.docx]
